# The anti-obesity effect of namodenoson, an A3 adenosine receptor agonist

**DOI:** 10.1038/s41366-026-02017-2

**Published:** 2026-02-15

**Authors:** Pnina Fishman, Inbal Itzhak, Rifaat Safadi, Johnny Amer, Ahmad Salhab, Avital Bareket-Samish

**Affiliations:** 1 Can-Fite BioPharma Ltd, Petah-Tikva, Israel; 2 Liver Institute, Hadassah Medical Organization, Jerusalem, Israel; 3 BioInsight Ltd, Binyamina, Israel

**Keywords:** Obesity, Obesity

## Abstract

Namodenoson—a selective A3 adenosine receptor (A3AR) agonist—is currently in clinical trials for hepatocellular carcinoma and metabolic dysfunction-associated steatotic liver disease. This preclinical study investigated its potential utility as a weight-loss drug. In 3T3-L1 adipocyte cells, namodenoson exhibited a dose-dependent inhibitory effect on the proliferation and accumulation of lipid droplets. Compared to vehicle, 5 and 10 nM of namodenoson inhibited adipocyte proliferation (determined using ^3^H-thymidine incorporation assay) by 26 ± 12% (*P* < 0.05) and 54 ± 5% (*P* < 0.001), respectively, and lipid accumulation (determined by Oil-Red-O staining) by 22 ± 8% (*P* < 0.05) and 41 ± 9% (*P* < 0.001), respectively. Western blot analyses using 3T3-L1 adipocyte cells demonstrated that namodenoson led to downregulation of A3AR, PPAR*γ*, C/EBP*α*, C/EBPβ, p-AKT, PI3K, NF-kB, and β-catenin, and upregulation of adiponectin. In-vivo experiments in a murine model of diet-induced obesity demonstrated that administering daily namodenoson (100 μg/kg) to high-fat-fed mice led to a significant difference in weight after 4 weeks of treatment compared to high-fat-fed mice without namodenoson (44.3 ± 2.2 vs 47.2 ± 3.4 g, respectively, *P* = 0.001), representing a difference in weight of 6.1%. The same experiment on mice fed a lean diet demonstrated no namodenoson effect (mean weight: 33.5 ± 3.9 vs 33.0 ± 0.6 g, respectively). In conclusion, our findings support continued investigation of namodenoson as a weight-loss drug candidate.

## Introduction

Obesity increases the risk for many diseases, including type 2 diabetes (T2DM), metabolic dysfunction-associated steatotic liver disease (MASLD), cardiovascular diseases, and certain cancers. The recently approved glucagon-like peptide-1 (GLP-1) receptor agonist medications treat obesity effectively; however, they have significant safety/tolerability issues [1, 2].

In obesity, lipid droplets accumulate in pre-adipocytes, which differentiate into mature adipocytes that secrete several adipokines, including adiponectin, which has insulin-sensitizing, angiogenic, anti-inflammatory, and vasodilatory properties. Adiponectin plays important protective roles in the cardiovascular and nervous systems and obesity-related diseases. Adiponectin levels were shown to be significantly lower in some insulin-resistant states, such as obesity and T2DM [3–6].

Namodenoson is an A3 adenosine receptor (A3AR) agonist, currently under clinical development for hepatocellular carcinoma and metabolic dysfunction-associated steatohepatitis (MASH) [7]. Namodenoson has a differential effect on cancer/inflammatory cells, which highly express A3AR, vs normal cells where A3AR expression is low. The binding of namodenoson to A3AR on cancer/inflammatory cells leads to inhibition of phosphoinositide 3 kinase (PI3K), subsequent deregulation of the Wnt/β-catenin and nuclear factor kappa B (NF-kB) pathways, and ultimately to anti-proliferation effects and apoptosis. In contrast, the effect of namodenoson on non-cancer/inflammatory cells, which some evidence suggests is mediated via pathways involving adiponectin, leads to protective effects in the central nervous system, cardiovascular system, and the liver [3–10] Indeed, in a MASH animal model and in the phase 2 clinical study in MASLD/MASH, namodenoson treatment was shown to induce an increase in serum adiponectin [9, 10].

We investigated namodenoson’s effect on adipocyte proliferation and lipid accumulation, and the molecular events involved in vitro, and its effect on fat loss in vivo.

## Materials and methods

Tissue culture media, serum, phosphate-buffered saline (PBS), and additives were purchased from Hyclone (South Logan, UT, USA). Murine pre-adipocytes (3T3-L1, American Type Culture Collection; Manassas, VA, USA) were maintained in Dulbecco’s modified Eagle’s medium (DMEM) plus 10% newborn calf serum and antibiotics. For adipocyte differentiation induction, pre-adipocytes were plated at 24-well plates. After confluence, the cells were incubated in DMEM plus 10% fetal bovine serum, 1 mM dexamethasone, 0.5 mM 3-iso-butyl-1-methylxantine (IBMX), and 1 mg/ml insulin for 2 days followed by treatment with insulin alone. The medium was replaced every 2 days for 8–14 days. 3T3-L1 cells (5000 cells/well) were incubated with 5 or 10 nM namodenoson (Can-Fite BioPharma Ltd, Petah Tikva, Israel) or vehicle at a 96-well plate for 48 h, and cell proliferation was assessed by the ^3^H-thymidine incorporation assay (Sanquine, Amsterdam, The Netherlands).

For lipid accumulation analysis, pre-adipocytes were incubated in the differentiated medium (high-glucose DMEM high with 10% fetal bovine serum and 10 mg/ml insulin, 1 mM dexamethasone and 0.5 mM IBMX), and the cells were treated with 5 or 10 nM namodenoson or vehicle for 48 h. Accumulation of lipid droplets was evaluated by Oil-Red-O staining (Sigma Aldrich, St. Louis, Missouri, USA). The cells were washed with PBS and incubated with 3.7% formaldehyde in H_2_O (Sigma Aldrich, St. Louis, Missouri, USA) for 1 h, followed by incubation with Oil-Red-O solution for 45 min. Cells were washed, visualized, and photographed under an Olympus microscope (Tokyo, Japan). Cells were dissolved in isopropanol to quantify lipid accumulation, and the optical density was read on a Dynatech Corp. Microelisa reader (Chantilly, VA, USA) at 595 nm. Experiments were performed in triplicates; data are presented as mean ± SD. Statistical significance was determined by t-test. *P* < 0.05 was considered statistically significant.

Western blot analyses were performed with culture conditions as in the ^3^H-thymidine experiments with 5 or 10 nM namodenoson or vehicle. After incubation for 24 h, the cells were rinsed with ice-cold PBS and transferred to ice-cold radio-immunoprecipitation assay buffer with 1× protease & phosphatase inhibitor cocktail (ThermoFisher Scientific, Waltham, MA, USA) for 20 min. Cell debris was removed by centrifugation at 4 °C for 10 min, at 7500 × *g*. The supernatant was used for Western blot analyses. Protein concentrations were determined using the NanoDrop assay (ThermoFisher Scientific, Waltham, MA, USA). Fifty micrograms were separated by 12% SDS-PAGE (ThermoFisher Scientific, Waltham, MA, USA), and electroblotted onto nitrocellulose membranes (Schleicher & Schuell, Keene, NH, USA). Membranes were blocked with 1% bovine serum albumin and incubated with the relevant primary antibodies (dilution 1:1000, all purchased from Santa Cruz Biotechnology, Dallas, TX, USA, catalog numbers are detailed in Supplementary Table 1) for 24 h at 4 °C. Blots were washed and incubated with the secondary antibody for 1 h at room temperature. Bands were recorded using a BCIP/NBT kit (Promega Madison, WI, USA).

All laboratory animal procedures were evaluated and approved by The Hebrew University Institutional Animal Care and Use Committee (approval #MD-21-16593-3) and followed the guidelines for laboratory animal welfare. Mice studies utilized C57BL/6 J males, 4–6 weeks old (The Jackson Laboratory, stock 000664, Bar Harbor, ME, USA). The mice (6 in each treatment group) were fed a high-fat diet (D12492, 60 kcal% fat; Research Diets, Inc., New Brunswick, NJ, USA) or a regular diet (“lean diet group”). After 12 weeks on this diet, mice received daily namodenoson (100 μg/kg, oral) or remained untreated. Weight was measured weekly from treatment initiation up to week 16. Data are presented as mean±SD. The difference in weight between the groups was determined by t-test. *P* < 0.05 was considered statistically significant.

## Results

**Fig. 1:**
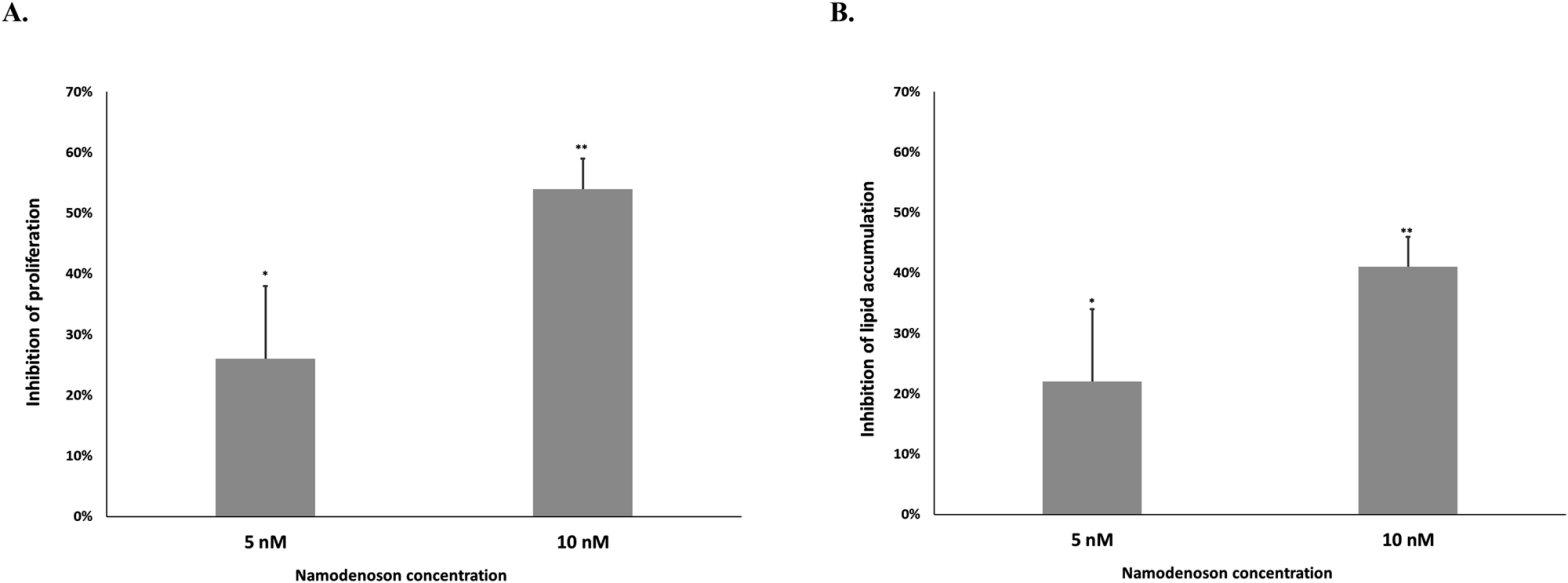
The effect of namodenoson on adipocyte proliferation and lipid accumulation. **A** Dose-dependent inhibition of adipocyte proliferation by namodenoson compared to control (cells treated with vehicle), as determined using the ^3^H-thymidine incorporation assay. The experiment was performed in triplicate. The bars represent the mean, and the error bars represent SD. *P < 0.05, **P < 0.001 (t-test). **B** Dose-dependent inhibition of lipid droplet accumulation in adipocyte treated with namodenoson compared to control (cells treated with vehicle), as determined by Oil-Red-O staining. The experiment was performed in triplicate. The bars represent the mean, and the error bars represent SD. *P < 0.05, **P < 0.001.

At 5 and 10 nM, namodenoson inhibited murine adipocyte (3T3-L1) proliferation by 26% ± 12% and 54% ± 5%, respectively, compared to the vehicle-treated cells. This dose-dependent effect was significant at both doses (*P* < 0.05 and *P* < 0.001, respectively) (Fig. 1A). Similarly, at concentrations of 5 and 10 nM, namodenoson inhibited lipid accumulation by 22 ± 8% and 41 ± 9%, respectively, compared to vehicle-treated cells. This dose-dependent effect was also significant at both doses (*P* < 0.05 and *P* < 0.001, respectively) (Fig. 1B).

**Fig. 2:**
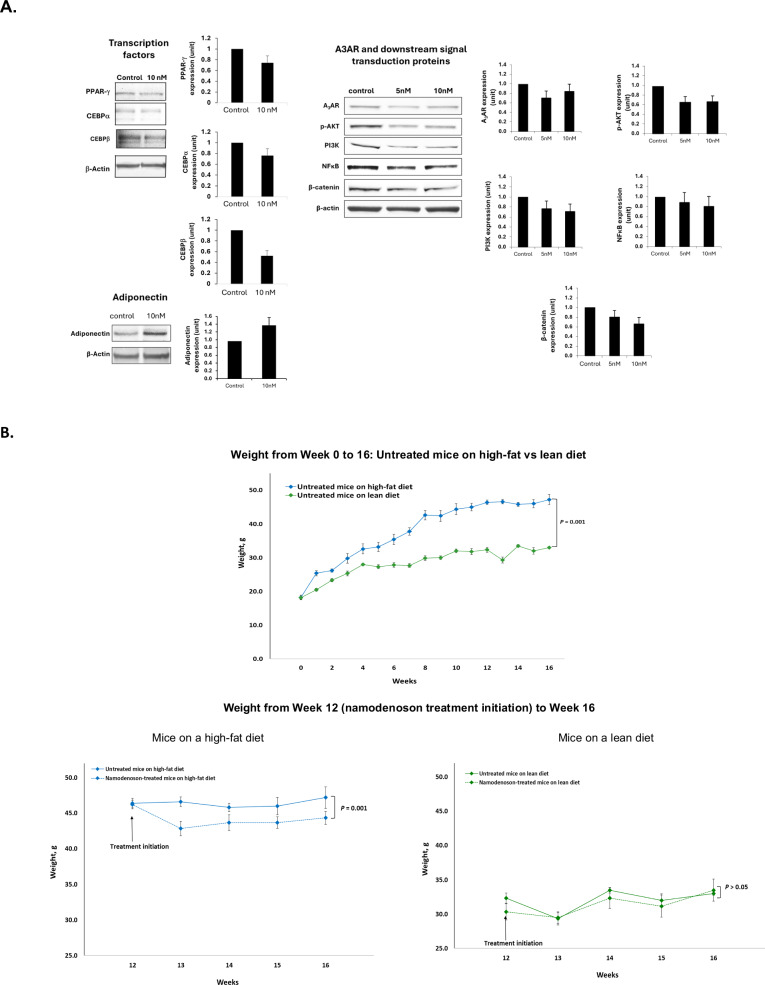
In-vitro and in-vivo analyses demonstrating the effects of namodenoson on adipocytes in cell culture and weight in a murine model of diet-induced obesity. **A** Western blot analysis results of 3T3-L1 adipocytes treated with namodenoson vs controls (cells treated with vehicle). The experiment was performed in triplicate. Protein levels were normalized to the controls; error bars represent SEM. **B** Analysis of mice weight over time. Top panel shows the change in weight over time in untreated mice on a high-fat versus lean diet; bottom panel shows the change in weight over time, from namodenoson treatment initiation at weeks 12–16 in mice on a high-fat diet (bottom left) or lean diet (bottom right) with and without namodenoson treatment. Each treatment group included 6 mice. Error bars represent SE. Statistical significance was determined using a t-test.

Treatment of 3T3-L1 adipocytes with namodenoson resulted in downregulation of peroxisome proliferator-activated receptor-*γ* (PPAR*γ*), CCAAT/enhancer binding protein (C/EBP)*α*, and C/EBPβ, known to act as transcription factors of adiponectin. Notably, upregulation of adiponectin was observed, whereas downregulation of A3AR, p-AKT, PI3K, NFκB, and β-catenin expression levels was noted (Fig. 2A).

The difference in weight between untreated mice fed high-fat diets and those fed lean diets increased over time. At week 16, untreated mice on a high-fat diet were heavier than those on a lean diet (mean, 47.2 ± 3.4 vs 33.0 ± 0.6 g). This difference was statistically significant (*P* = 0.0001) (Fig. 2B). Following the administration of daily namodenoson (oral, 100 μg/kg) to the high-fat-fed mice starting at week 12, a significant difference in weight was noted after 4 weeks (i.e., at week 16) compared to high-fat-fed mice who did not receive namodenoson (mean, 44.3 ± 2.2 vs 47.2 ± 3.4 g, respectively, *P* = 0.001). This difference represents a difference in weight of 6.1%. The same experiment performed on mice fed with lean diet demonstrated no effect for namodenoson (mean, 33.5 ± 3.9 vs 33.0 ± 0.6 g) (Fig. 2B).

## Discussion

This preclinical study provides the first evidence for the potential utility of namodenoson as a weight-loss treatment by demonstrating that namodenoson inhibited the proliferation of adipocytes and the accumulation of lipid droplets, and that namodenoson led to weight loss in diet-induced obesity in mice. The preliminary mechanism suggested by our findings involved namodenoson-induced upregulation of adiponectin.

Our in-vitro findings are consistent with a prior preclinical study evaluating the effect of namodenoson in a MASH mouse model, where namodenoson treatment was associated with a statistically significant upregulation of adiponectin [10]. Our findings are also consistent with the increase in serum adiponectin levels observed in patients with MASLD/MASH who were treated with namodenoson in a phase 2 study. Furthermore, in this phase 2 trial, dose-dependent weight loss over time was also demonstrated [9].

Literature evidence suggests a link between TNF-α and adiponectin, whereby inhibition of the former leads to upregulation of the latter [11]. Ours and other authors’ findings demonstrate inhibition of NF-kB and direct TNF-α inhibition via A3AR agonists [12–14]. It may be assumed that the upregulation of adiponectin shown in the current study is mediated through this pathway, which is also supported by Merino et al., who documented the role of A3AR in stimulating adiponectin production in white adipose tissue [15]. PPAR*γ*, C/EBP*α*, C/EBPβ, and NF-kB are among other transcription factors responsible for adiponectin production and expression. In the current study, we noted that all 4 transcription factors were down-regulated upon treatment of 3T3-L1 cells with namodenoson, in parallel to the increase in the expression level of adiponectin. This can be explained by data showing that adiponectin acts as an anti-inflammatory hormone and suppresses the activation of NF-kB and other inflammatory transcription factors responsible for inflammatory cytokine transcription. Namodenoson has been shown to exhibit hepatoprotective, cardioprotective, and neuroprotective effects, which may be mediated through adiponectin [3–7, 9, 10]. The current study suggests that namodenoson’s positive metabolic effect may also be adiponectin-mediated.

Unlike current GLP-1 receptor agonists, namodenoson is an oral drug, with an excellent safety profile as demonstrated in clinical trials. This safety profile is probably related to its cardio- neuro- and hepato- protective effects [3–7, 9, 10, 16].

In conclusion, our findings, along with the body of preclinical and clinical evidence regarding namodenoson, suggest that namodenoson has potential utility as a weight-loss drug.

## Supplementary information

Supplementary Materials

## Data Availability

The data that support the findings of this study are available from the corresponding author upon reasonable request.
