# Supplementary material for: The anti-obesity effect of namodenoson, an A3 adenosine receptor agonist
Source: Int J Obes (Lond). 2026 Feb 15;50(4):869–72. doi: 10.1038/s41366-026-02017-2 (PMC13056519; doi:10.1038/s41366-026-02017-2)
Supplement: Supplementary file 1 — Supplementary Materials [file 41366_2026_2017_MOESM1_ESM.pdf]

Supplementary Materials

Supplementary Table 1. List of primary monoclonal antibodies used in the Western Analyses

#	**Antibody target**	**Santa Cruz Biotechnology catalog number**
1	A3AR	sc-13938
2	Adiponectin	sc-136131
3	p-AKT	sc-514032
4	β-actin	sc-47778
5	β-catenin	sc-7963
6	C/EBPα	sc-365318
7	C/EBPβ	sc-7962
8	NFκB	sc-8008
9	PI3K	sc-377482
10	PPARγ	sc-7273
